# Long working hours and risk of cardiovascular outcomes and diabetes type II: five-year follow-up of the Gutenberg Health Study (GHS)

**DOI:** 10.1007/s00420-021-01786-9

**Published:** 2021-11-12

**Authors:** Rossnagel K, Jankowiak S, Liebers F, Schulz A, Wild P, Arnold N, Seidler A, Hegewald J, Romero Starke K, Letzel S, Riechmann-Wolf M, Nübling M, Beut-el M, Pfeiffer N, Lackner K, Münzel T, Poplawski A, Latza U

**Affiliations:** 1 Federal Institute for Occupational Safety and Health, Bundesanstalt für Arbeitsschutz und Arbeitsmedizin (BAuA), Nöldnerstr. 40-42, 10317 Berlin, Germany; 2 University Medical Center of the Johannes Gutenberg University of Mainz, Mainz, Germany; 3 Institute and Polyclinic of Occupational and Social Medicine (IPAS), Carl Gustav Carus Faculty of Medicine, TU Dresden, Dresden, Germany; 4 Institute of Sociology, Faculty of Behavioral and Social Sciences, TU Chemnitz, Chemnitz, Germany; 5 Institute of Occupational, Social, and Environmental Medicine, University Medical Center of the Johannes Gutenberg University of Mainz, Mainz, Germany; 6 FFAW: The Freiburg Research Centre for Occupational Sciences, Freiburg, Germany

**Keywords:** Prospective cohort study; cardiovascular disease, Diabetes, Arterial stiffness, Occupational health, Working time

## Abstract

**Objectives:**

The aims of this study were to determine if there was an increased risk of incident cardiovascular disease (CVD) and diabetes and an increase in arterial stiffness in participants who reported working 41–54 h per week and more than 55 h compared to those who worked 40 h or less over a time interval of 5 years.

**Methods:**

In a subsample of the population-based prospective Gutenberg Health Study (GHS) study, we examined working participants younger than 65 years at baseline (*n* = 7241) and after 5 years. To test the association of working time at baseline and incident cardiovascular events and diabetes type II, we estimated hazard ratios (HR) using competing risks models. For a change in the arterial stiffness index (SI) based on assessment using a Pulse Trace PCA2 device, we used multivariate linear regression models.

**Results:**

The SI increased in those working more than 55 h per week (beta coefficiant = 0.32 m/s (95% CI 0.07–0.58) compared to those working 40 h and less after adjustment for sex, age and SES. Due to small numbers there was no significant association of working hours and clinically manifest cardiovascular events and diabetes type II in the 5-year follow-up time.

**Conclusions:**

Further studies are needed to confirm the results on working hours and arterial stiffness. Analyses of the 10-year follow-up with more events may clarify the results for incident cardiovascular events and metabolic outcomes.

**Supplementary Information:**

The online version contains supplementary material available at 10.1007/s00420-021-01786-9.

## Introduction

A number of studies have suggested that long working hours may have adverse effects on health in general (Bannai and Tamakoshi 2014). However, results have been diverse when looking at cardiovascular diseases (CVD). Whereas meta-analyses (Kivimäki et al. 2015a; Virtanen and Kivimäki 2018; Virtanen et al. 2012) and studies in Asian countries (Imai et al. 2014; Shin et al. 2017) showed an association of long working hours with CVD, recent studies did not find an association in European countries (Hannerz et al. 2018a, 2018b, Alicandro et al. 2020). There is less conclusive data for the association of diabetes with long working hours (Kivimäki et al. 2015b).

A major limitation among the underlying studies of the meta-analyses is the inconsistent assessment of the exposure “long working hours”. Some studies have used reported overtime work in general, while others have assessed daily working hours or weekly working hours with different cut-off points. The most comprehensive meta-analysis suggests a 1.12-fold (95% CI 1.03–1.21) increased risk associated with coronary heart disease and a 1.21-fold (95% CI 1.01–1.45) increased risk of stroke for those working ≥ 55 h per week (Virtanen and Kivimäki 2018). A systematic review that looked at type II diabetes as outcome showed a weaker association (Kivimäki et al. 2015b); the minimally adjusted RR of diabetes for long (≥ 55 h per week) compared with standard working hours was 1.07 (95% CI 0.89–1.27) in 222,120 individuals. A significant association between long working hours and diabetes was evident only in the lowest socio-economic status group with a relative risk (RR) of 1.29 (95% CI 1.06–1.57).

In 2019, the EU average working week consisted of 37.0 h. The longest average working week was found in Greece (41.7 h per week) and the shortest in the Netherlands (30.4 h) with Germany at the lower end with 34.8 h (Eurostat 2019). A recent survey of full-time employees in Germany found that 21% worked between 40 and 48 h and 15% more than 48 h (BAuA, 2020). That number has remained stable over the last 12 years.

Employees working long hours may be more exposed to psychosocial hazards (stress such as high demands) and physical workplace hazards (noise, chemicals, lack of natural light, etc.) (Virtanen and Kivimäki 2018, Girard et al. 2015). Prolonged sitting at the workplace could also have adverse effects (Ferrario et al. 2019*).* On the other hand, employees working long hours may have reduced time available for other activities besides work such as physical and social activities, relaxation, sleep, etc. (Garthus-Niegel et al. 2016). In addition, the pattern of breaks and relaxation times during working hours may play a role (Backhaus et al. 2019).

The aim of our study was to analyse in detail the exact working hours for each participant via interview and to report the event of cardiovascular events and diabetes type II 5 years later. There are a number of subclinical markers related to the risk of cardiovascular events, among them arterial stiffness. Digital photoplethysmography utilises an infrared light to measure the volumetric variations of blood circulation and was validated in GHS baseline (Arnold et al. 2017). It represents an easily performable and operator-independent alternative technique to measure arterial stiffness compared to the standard pulse wave velocity (PWV) (Townsend et al. 2015). To our knowledge, this method has not been used in another large population-based study before. In the cross-sectional analysis of the GHS baseline, work in the night shift was associated with a significantly increased arterial stiffness (Jankowiak et al. 2016).

Study questions.

1) Is the risk of developing a cardiovascular event and the occurrence of clinically manifested diabetes type II higher in employees who report working 41–54 h and more than 55 h per week compared to those who work 40 h or less over a time interval of 5 years?

2) As the time interval is relatively short, is there a larger increase in arterial stiffness as a subclinical marker in the two groups that work more than 40 h per week compared to those working 40 h or less?

## Methods

### Design and participants

A total of *N* = 15.010 participants were enrolled in the Gutenberg Health Study (GHS) between the years 2007 and 2012. The Gutenberg Health Study is a German population-based, prospective, single-centre cohort study in the Rhine-Main-Region. The primary aim of the study is to analyse and improve cardiovascular risk factors. The local ethics committee and the local and federal data safety commissioners have approved the study procedure (reference number 837.020.07(5555)). The participants were determined randomly from the local registry of the city of Mainz and of the district of Mainz-Bingen. The sample strategy considered sex, residence and age. Inclusion criteria for the GHS were having a written informed consent and age between 35 and 74 years. Persons with insufficient German language knowledge were not included in the study, as well as persons who were not able to visit the study centre due to physical and/or mental impairment. A detailed description of the design and the rationale of the GHS has been published elsewhere (Wild et al. 2012).

**Fig. 1:**
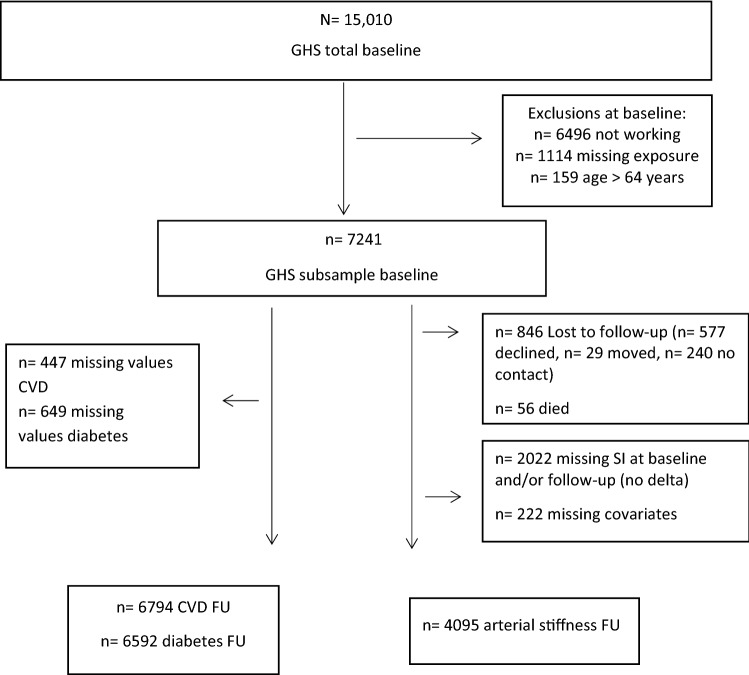
Flow chart of study population the Gutenberg Health Study (GHS) for the analysis on long working hours Legend: CVD= cardiovascular disease, SI= Stiffness Index

For the present analysis, 6496 participants were not eligible because they did not work. We excluded those older than 64 years of age (*n* = 159) at baseline and those participants with missing exposure (working time) (*n* = 1114) leaving 7241 participants for baseline analysis. For the incident events of CVD and occurrence of diabetes mellitus type II, participants with these events at baseline were excluded. The detailed flow chart is shown in Fig. 1.

### Data assessment

At baseline and follow-up the participants underwent a 5-h assessment in the study centre of the GHS. The information collected during the examination comprised a computer-assisted interview including questions about lifestyle, patient history regarding illness and medication. In addition clinical and laboratory parameters (venous blood sample), blood pressure and anthropometric measurements were recorded. All tests were conducted fulfilling standard procedures by certified staff.

### Outcomes

A cardiovascular event was defined as a main diagnosis for first acute myocardial infarct (ICD-10: I21), sudden cardiac death (I46), cerebral infarction/ischemic stroke (I63) or coronary artery disease (I25.10). A team of experts (endpoint committee) validated and confirmed each CVD event retrospectively. Life status was checked for every subject not participating in the follow-up examination (FU). Deaths of participants in the follow-up period due to other reasons not caused by CVD were marked as right censored.

Hospital records, information from attending physicians and study participants about all relevant illnesses including diabetes were obtained and evaluated by the so-called endpoint committee of the GHS. In the case of death, the death certificate was obtained from the local health authorities (“Gesundheitsamt”). The committee consisted of two physicians and an epidemiologist. During regular meetings, the committee assessed the events and categorized them in terms of the definition of endpoints set in the study protocol.

Diabetes mellitus type II was defined as either a measured HbA1c-level ≥ 6.5%, intake of anti-diabetic drugs (ATC code A10), or answering yes to the question in the interview “Has your diabetes been diagnosed by a physician?”.

Arterial stiffness was assessed using the Pulse Trace PCA2 device (Micro Medical Limited/Carefusion) at baseline and FU. This device uses digital photoplethysmography, which transmits an infrared light at 940 nm through the finger. The amount of absorbed light is proportionally related to the volume of blood in the finger pulp (Arnold et al. 2017). The stiffness index (SI) was measured in meter per seconds (m/s) (body height/peak-to-peak time).

### Exposure

#### Total working time

Participants were asked via a questionnaire what their regular hours per week were and how many hours per week, they worked overtime for their current job and past jobs (maximum of 15 jobs inquired in job history). Total working time was defined as regular (fixed) working hours plus overtime at the current job at baseline.

### Covariates

#### General

Age was treated as a categorical variable with categories of decades: 35–44 years, 45–54 years and 55–64 years. Socio-economic status (SES) was used as an index score comprising school education, professional education, occupational position, and salary (Lampert and Kroll 2009). The scale ranges from 3 to 21 points. For the descriptive visualisation of the population, the empirically recommended cut points of < 7.8 (low SES), 7.8–14 (intermediate SES), and > 14 (high SES) were used (Lampert et al. 2006).

#### Lifestyle factors

Smoking was dichotomised into smokers (occasional smokers and smokers) and non-smokers (never smokers and ex-smokers). To estimate pack years, the smoking history was enquired including its duration and the type of tobaccos (filter tips, cigarettes, cigars, tobacco, and pipe). An alcohol intake > 10 g/day for women and for men an intake > 20 g per day was defined as an intake above the tolerable limit. Anthropometric measurements were taken with calibrated digital scales (Seca 862, Seca, Hamburg, Germany), a measuring stick (Seca 220, Seca, Hamburg, Germany) and a non-stretching waist measuring tape. Waist circumference was measured midway between the lower rib margin and the superior anterior iliac spine in cm. Physical activity was assessed by the SQASH score (Campbell et al. 2016).

#### Occupational factors

Occupations were manually double-coded according to the classification of occupations of the Federal Statistical Office Germany (KldB 2010). The KldB 2010 is coded in five digits and is hierarchically structured as described earlier (Prigge et al. 2012). The first digit of the code describes the occupational area, the second the main occupational group, the third the occupational group, the fourth the occupational subgroup (fourth digit = 9 and some special KldB2010 codes defining managerial and supervisor position), and the fifth job complexity. Job complexity contains four levels: “low” (helpers), “medium” (skilled workers), “complex” (specialists) and “very complex” (experts). Other questions included type of employment (part-time/full-time employment, self-employment/employee) and night shift (yes/no) and years at the current workplace.

### Statistical analysis

Descriptive analyses were carried out for the sample stratified by sex and total working time.

Time to event analysis: To test the association of working time at baseline and incident cardiovascular/metabolic outcomes we estimated hazard ratios (HR) and cumulative incidence functions using the Fine and Gray subdistribution hazards model (Fine and Gray 1999). The number of years after baseline examination to the first occurrence of a confirmed CVD event defined time to event. Events that were non-CVD deaths were defined as competing events. Participants who discontinued the study due to reasons not related to CVD were right censored.

For the change in arterial SI from baseline to follow-up we used multivariate linear regression models. Absolute changes in the SI were estimated using linear regression models where the dependent variable was the delta baseline to FU SI value. In addition, linear models with SI at t_0_ as off-set were calculated facilitating the interpretation of the absolute delta in SI. The scale of SI remained the same in both models [m/s].

Five different adjustment sets for all outcomes were defined a-priori. Model 0: crude model (exposure only), Model 1: sex and age, Model 2: model 1 plus night shift (yes/no), managerial/supervisor position (yes/no, derived from KldB2010), years at a current workplace; Model 3: model 1 plus waist to height ratio, smoking status (yes/no), pack-years, alcohol consumption (above tolerable limit yes/no), physical activity (SQASH), menopausal status (yes/no) Model 4: model 1 plus SES, Model 5: all confounders. SES was entered separately because it is linked with managerial/supervisor position. A p-value < 0.05 was considered as significant.

All analyses were conducted using the R version 4.0.3 (2020) software package.

## Results

### Descriptive analysis

Of the 15,010 overall study participants, a total of 7241 were eligible for analysis at baseline (Fig. 1). Mean age was 48.3 years and 46% were female. In total 3459 participants (48%) reported working forty hours or less, 2906 participants (40%) between over 40 and under 55 h and 876 participants (12%) 55 h or more. As expected for the German working population women were more likely to work part-time than men (45.3% vs. 3.6%) and few women worked 55 h or more (*n* = 164) compared to men (*n* = 712). The baseline sample characteristics for the participants included in the analysis of incident CVD, occurrence of diabetes and change in SI are presented in Tables 1a and 1b.

**Table 1:** Sample characteristics of the analysis sample of the Gutenberg Health Study (GHS) Characteristics by sex (*n* = 7241)

	All (*n* = 7241)	Women (*n* = 3332)	Men (*n* = 3910)	*p* for trend
Age [y] (mean ± SD)	48.3 ± 7.6	48.1 ± 7.4	48.6 ± 7.7	*
*General*				
Qualification for university	3445 (47.6%)	1433 (43.0%)	2012 (51.5%)	n.a
SES (mean ± SD)	14.07 ± 4.20	13.53 ± 3.92	14.53 ± 4.38	***
*Anthropometrics*				
WHtR (mean ± SD)	0.54 ± 0.08	0.53 ± 0.08	0.55 ± 0.07	***
Still regular period	-	1682 (50.6%)	-	-
Menopausal age [y] (mean ± SD)	-	46.82 ± 6.31	-	-
*Life style*				
Smoking	1731 (23.9%)	788 (23.6%)	943 (24.1%)	n.sig
-pack-years (median Q1/Q3)	0.2 (0/3.6)	0.1 (0/2.7)	0.5 (0/4.3)	***
Alcohol per day [g] (median (Q1/Q3))	5.0 (0/16.9)	0 (0/9.4)	8.4 (0/22.0)	***
-Intake above tolerable limit	1698 (23.5%)	734 (22.0%)	964 (24.7%)	n.a
Activity score (mean ± SD)	8.4 ± 3.4	8.1 ± 3.0	8.7 ± 3.7	***
*Occupational factors*				
Years at current work place	14.05 ± 10.44	13.04 ± 10.25	14.90 ± 10.53	***
Managerial position	1085 (15.0%)	287 (8.6%)	798 (20.4%)	***
Self-employed	1059 (14.6%)	378 (11.4%)	681 (17.4%)	***
Employee	6178 (85.4%)	2951 (88.6%)	3227 (82.6%)	***
Full-time work	5591 (77.2%)	1821 (54.7%)	3770 (96.4%)	***
Part time employment	1650 (22.8%)	1511 (45.3%)	139 (3.6%)	***
Night shift	964 (13.3%)	254 (7.6%)	710 (18.2%)	***
Low job complexity°	261 (3.6%)	183 (5.5%)	78 (2.0%)	***
Medium job complexity°	3289 (45.4%)	1808 (54.3%)	1481 (37.9%)	***
High job complexity°	1524 (21.0%)	603 (18.1%)	921 (23.6%)	***
Very high job complexity°	2167 (29.9%)	738 (22.1%)	1429 (36.6%)	***
Working time total [h/w] (mean ± SD)	40.8 ± 13.0	34.5 ± 12.8	46.2 ± 10.6	***
(median (Q1/Q3))	41 (35/48)	36 (24/42)	45 (40/50)	***
Fixed working time [h/w] (mean ± SD)	36.9 ± 11.3	31.4 ± 11.1	41.6 ± 9.2	***
Overtime [h/w] (mean ± SD)	3.9 ± 6.1	3.10 ± 5.29	4.63 ± 6.56	***
Overtime > 20%	1265 (17.6%)	451 (13.7%)	814 (20.9%)	***
*CVRF*				
Obesity	1607 (22.2%)	663 (19.9%)	944 (24.1%)	***
FH of MI or Stroke	2358 (32.6%)	1143 (34.3%)	1215 (31.1%)	***
*S*tiffness-Index [m/s] (mean ± SD)	7.25 ± 2.13	6.39 ± 1.57	7.95 ± 2.27	***

Characteristics by total working time (*n* = 7241)					
	All (*n* = 7241)	≤ 40 h/w] (*n* = 3459)	41–54 h/w (*n* = 2906)	≥ 55 h/w (*n* = 876)	*p* for trend
Sex (Women)	3332 (46.0%)	2269 (65.6%)	899 (30.9%)	164 (18.7%)	***
Age [y] (mean ± SD)	48.3 ± 7.6	48.6 ± 7.5	47.9 ± 7.6	49.0 ± 7.7	n.sig
*General*					
Qualification for university	3445 (47.6%)	1410 (40.8%)	1558 (53.6%)	477 (54.5%)	n.a
SES (mean ± SD)	14.07 ± 4.20	13.05 ± 4.06	14.85 ± 4.07	15.53 ± 4.19	***
*Anthropometric*					
WHtR (mean ± SD)	0.54 ± 0.08	0.54 ± 0.08	0.54 ± 0.07	0.55 ± 0.07	**
*Life style*					
Smoking	1731 (23.9%)	829 (24.0%)	673 (23.2%)	229 (26.1%)	n.sig
-pack-years (median (Q1,Q3))	0.2 (0/3.6)	0.1 (0/3.3)	0.3 (0/3.5)	0.4 (0/5.2)	*
Alcohol per day (g) (median (Q1,Q3))	5.0 (0/16.8)	2.5 (0/12.6)	5.6 (0/18.9)	7.54 (0/20.6)	***
-Intake above tolerable limit	1698 (23.5%)	768 (22.2%)	708 (24.4%)	222 (25.3%)	n.a
Activity score (SQUASH) (mean ± SD)	8.4 ± 3.4	8.0 ± 3.2	8.5 ± 3.3	9.7 ± 4.3	***
*Occupational factors*					
Years at current work place	14.1 ± 10.4	13.5 ± 10.5	14.4 ± 10.2	15.0 ± 10.9	***
Managerial Position	1085 (15.0%)	228 (6.6%)	557 (19.2%)	300 (34.2%)	***
Self-employed	1059 (14.6%)	356 (10.3%)	295 (10.2%)	408 (46.6%)	***
Employee	6178 (85.4%)	3100 (89.7%)	2611 (89.8%)	467 (53.4%)	***
Full-time	5591 (77.2%)	1857 (53.7%)	2866 (98.6%)	868 (99.1%)	***
Part time employment	1650 (22.8%)	1602 (46.3%)	40 (1.4%)	8 (0.9%)	***
Night shift	964 (13.3%)	328 (9.5%)	412 (14.2%)	224 (25.6%)	***
Low job complexity^a^	261 (3.6%)	218 (6.3%)	34 (1.2%)	9 (1.0%)	***
Medium job complexity^a^	3289 (45.4%)	1921 (55.5%)	1099 (37.8%)	269 (30.7%)	***
High job complexity^a^	1524 (21.0%)	604 (17.5%)	724 (24.9%)	196 (22.4%)	***
Very high job complexity^a^	2167 (29.9%)	716 (20.7%)	1049 (36.1%)	402 (45.9%)	***
Working time total [h/w] (mean ± SD)	40.8 ± 13.0	31.1 ± 9.0	45.7 ± 3.4	63.1 ± 9.6	***
(median (Q1/Q3))	41 (35/48)	34 (24/39.5)	45 (43/50)	60 (57/65)	
Fixed working time [h/w] (mean ± SD)	36.9 ± 11.3	29.8 ± 9.3	40.6 ± 3.8	52.7 ± 13.2	***
Overtime [h/w] (mean ± SD)	3.9 ± 6.1	1.3 ± 2.7	5.1 ± 4.0	10.5 ± 12.0	***
Overtime > 20%	1265 (17.6%)	201 (5.9%)	692 (23.9%)	372 (43.7%)	***
*CVRF*					
Obesit	1607 (22.2%)	760 (22.0%)	627 (21.6%)	220 (25.1%)	n.sig
FH of MI or Stroke	2358 (32.6%)	1156 (33.4%)	926 (31.9%)	276 (31.5%)	n.sig
*S*tiffness-Index [m/s] (mean ± SD)	7.25 ± 2.13	6.94 ± .95	7.44 ± 2.20	7.82 ± 2.38	***

^a^ from KldB2010, n.sig = non significant, n.a. = not applicable

* =  < 0.05

** =  < 0.001,

^***^ =  < 0.0001, WHtR = Waist to height ratio, SES = social-economic status, h/w = hours per week, CVRF = cardiovascular risk factors, FH = family history, MI = myocardial infarction

### Incidence of CVD and occurrence of diabetes

**Fig. 2:**
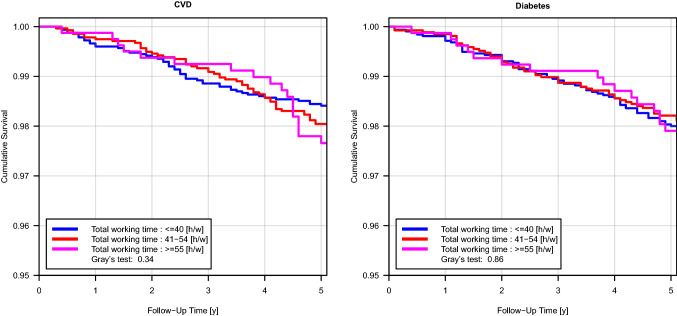
Time to event analysis for the incidence of (2.1) CVD and (2.2) diabetes

In total 6794 participants were analysed for CVD incidence. Altogether, 122 incident cardiovascular events and 34 competing events (non-CVD deaths) occurred among the analysed subgroup during the five-year follow-up (Table 2). The unadjusted hazard ratio (HR) was 1.19 (95% CI 0.81–1.76) for the intermediate group (41–54 h) and 1.45 (95% CI 0.85–2.47) for the group with the longest working hours compared to the reference group (supplementary Table S 1). Controlling for sex and age those numbers were 0.86 (0.58–1.29) and 0.86 (0.49–1.50), respectively. Adjusting for lifestyle factors, occupational factors and SES resulted in similar risk estimates. In Fig. 2 cumulative incidence plots with consideration of competing risk are shown.

**Table 2:** Associations of working time with an incidence of CVD and diabetes (time to event). Number of events, censored events, person-time and unadjusted incidence rates

	n at baseline	n at FU	Events within FU period	Censored events	Person years (py)	Incidence rate per 1000 py (95%Cl)
CVD						
All participants	7241	6816	122	6694	33,523	3.64 (3.02–4.35)
≤ 40 h/w	3459	3252	51	3201	16,007	3.19 (2.37–4.19)
41–54 h/w	2906	2765	53	2712	13,614	3.89 (2.92–5.10)
≥ 55 h/w	876	799	18	781	3902	4.61 (2.73–7.30)
Men	3909	3625	98	3527	17,726	5.53 (4.49–6.74)
≤ 40 h/w	1190	1081	34	1047	5251	6.47 (4.48–9.05)
41–54 h/w	2007	1898	48	1850	9326	5.15 (3.79–6.82)
≥ 55 h/w	712	646	16	630	3149	5.08 (2.90–8.23)
Women	3332	3191	24	3167	15,797	1.52 (0.97–2.26)
≤ 40 h/w	2269	2171	17	2154	10,755	1.58 (0.92–2.53)
41–54 h/w	899	867	5	862	4289	1.17 (0.38–2.72)
≥ 55 h/w	164	153	2	151	753	2.66 (0.32–9.60)
Diabetes						
All participants	7241	6613	126	6487	32,462	3.88 (3.23–4.62)
≤ 40 h/w	3459	3157	62	3095	15,511	4.00 (3.06–5.12)
41–54 h/w	2906	2669	48	2621	13,116	3.66 (2.70–4.85)
≥ 55 h/w	876	787	16	771	3835	4.17 (2.38–6.78)
Men	3909	3504	80	3424	17,129	4.67 (3.70–5.81)
≤ 40 h/w	1190	1039	30	1009	5055	5.93 (4.00–8.47)
41–54 h/w	2007	1833	35	1798	8997	3.89 (2.71–5.41)
≥ 55 h/w	712	632	15	617	3078	4.87 (2.72–8.04)
Women	3332	3109	46	3063	15,333	3.00 (2.20–4.00)
≤ 40 h/w	2269	2118	32	2086	10,456	3.06 (2.10–4.32)
41–54 h/w	899	836	13	823	4119	3.16 (1.68–5.40)
≥ 55 h/w	164	155	1	154	757	1.32 (0.03–7.36)

For diabetes, 126 events and 46 competing events occurred during the 5-year follow-up (Table 2). The unadjusted hazard ratio (HR) was 0.94 (95% CI 0.64–1.38) for the intermediate group (41–54 h) and 1.08 (95% CI 0.62–1.87) for those working 55 h and more compared to the reference group (supplementary Table S 2). Controlling for age and sex those numbers were 0.82 (95% CI 0.54 -1.23) and 0.84 (95% CI 0.48–1.49), respectively, resulting in no significant differences. Figure 2 shows the cumulative incidence plots. The number of both CVD and diabetes events was too small to make any robust conclusions.

### Stiffness index

Due to several exclusions (see Fig. 1) 4095 participants were analysed for change in arterial stiffness. In general, the 5-year changes in the SI increased with more working hours. Sex and age-adjusted beta coefficients were 0.04 m/s (95% CI -0.13–0.21) for the intermediate working hour group and 0.22 m/s (95% CI -0.03 -0.47) for those working 55 h or more (Table 3). In the latter group, increase in stiffness index was also non-significant when additionally adjusted for lifestyle and work factors (model 2 and 3), but marginally statistically significant results were observed when additionally adjusted for SES and “all-in” (model 4 und 5).

**Table 3:** Beta coefficients and 95% confidence intervals (CI) of arterial stiffness according to weekly working hours (h/w)

	Beta coefficients and 95% confidence intervals (CI) of arterial stiffness according to weekly working hours		
	≤ 40 h/w (Reference)	41–54 h/w	≥ 55 h/w
Model 0	1	0.52 (0.35–0.68)	0.90 (0.66–1.15)
Model 1	1	0.04 ( − 0.13–0.21)	0.22 ( − 0.03–0.47)
Model 2	1	0.05 ( − 0.12–0.23)	0.22 ( − 0.04–0.47)
Model 3	1	0.07 ( − 0.10–0.24)	0.23 ( − 0.03–0.47)
Model 4	1	0.11 ( − 0.07–0.28)	0.32 (0.07–0.58)
Model 5	1	0.11 ( − 0.07–0.28)	0.28 (0.02–0.54)

Model 0: crude model (exposure only); Model 1: sex and age; Model 2: model 1 plus night shift, managerial/supervisor position, years at current work place; Model 3: model 1 plus waist to height ratio, smoking status, pack-years, alcohol consumption, physical activity, menopausal status; Model 4: model 1 plus SES; Model 5: all confounders

Employing linear regression models with SI at t_0_ as an offset variable, results were similar (supplement S3). Beta coefficients adjusted for age and sex were 0.05 m/s (95% CI -0.13, 0.23) for the intermediate group and 0.20 m/s (95% CI -0.06, 0.47) for the group with the highest working hours, respectively. Further adjustment for SES resulted in similar numbers and did not reach statistical significance. Delta SI was, therefore, 1.05 times higher (5%) in the group 41–54 h per week and 1.20 times (20%) higher in the ≥ 55 h/week group than the reference group over a period of 5 years (non significant).

## Discussion

In this population-based cohort study, an increase of 0.32 m/s in the SI was detected for employees working ≥ 55 h/week during the 5-year FU. In the time to event analyses numbers for CVD and diabetes were too small to detect significant results.

### Comparison with other studies

The relatively modest effects of long working hours on the incidence of CVD in other studies are in line with the presented negative findings presumably due to a small number of events and with the indicative findings from the time to event analysis. In our study, the adjusted incidence rate for CVD was 0.86 with a wide confidence interval (95% CI 0.58–1.29) for those in the highest working hours group compared to the reference group. In a recently updated meta-analysis working ≥ 55 h/week increased the risk of IHD incidence compared with working 35–40 h/week (RR 1.13, 95% CI 1.02 -1.26) and for IHD mortality the RR was 1.17 (95% CI 1.05 -1.31) (Li et al. 2020). Within the framework of the World WHO/ILO Joint Estimates of the Work-related Burden of Disease and Injury, evidence on exposure to working ≥ 55 h/week was judged “as sufficient evidence of harmfulness for IHD incidence and mortality” (Li et al. 2020). Virtanen and Kivimäki (2018) report very similar results, namely a 1.12-fold (95% CI 1.03–1.21) increased risk associated with coronary heart disease.

For the outcome stroke, the results are more varied; Virtanen and Kivimäki report a 1.21-fold (95% CI 1.01–1.45) increased risk of stroke for those working ≥ 55 h per week (Virtanen and Kivimäki 2018). In the joint WHO/ILO publication the risk of acquiring a stroke was 1.35 (95% CI 1.13–1.61) of those working ≥ 55 h per week compared with working 35–40 h/week. When looking at stroke mortality the relative risk was 1.08 (95% CI 0.89–1.31) for this group. Evidence on exposure to ≥ 55 h/week was judged as “sufficient evidence for harmfulness for stroke incidence” and “inadequate evidence for harmfulness” for stroke mortality (Descatha et al. 2020). Due to small numbers, we could not analyse stroke incidence separately.

The even weaker effects of long working hours on the incidence of diabetes in other studies are also in line with the even more indicative findings from the time-to-event analysis. Kivimäki et al. (2015b) showed only a weak non-significant association for the outcome diabetes; the minimally adjusted RR of diabetes for long (≥ 55 h per week) compared with standard working hours was 1.07 (95% CI 0.89–1.27). In our study, the adjusted incidence rate for diabetes was 0.82 with a wide confidence interval (95% CI 0.54–1.23) for those in the highest working hours group.

In order to take into account the relatively short time of follow-up, we chose the subclinical marker of arterial stiffness as a further outcome. Arterial stiffening is being recognised as a critical precursor of cardiovascular disease (Mitchell et al. 2010). In our study working ≥ 55 h per week was associated with an increased SI (beta = 0.22 (95% CI -0.03–0.47)) compared to those working 40 h or less. The difference increased after additional adjustment for occupational factors and lifestyle, and was significant when adjusting for SES (beta = 0.32 (0.07–0.58)). When we stratified these results by gender, beta coefficients were similar for men as there was a significant difference in Model 4 und 4 for men working 55 h and more compared to the reference group (data not shown). For women, no significant association was found in any of the models. The interpretation, however, is difficult as few women worked 55 h and more and it remains to be seen whether this holds true if more women can be included in this group.

### Strengths and Limitations

The main strength of this study is its prospective nature and the fact that CVD events were based on medical records and confirmed by a committee of experts. Arterial stiffness index (SI) as an objective subclinical marker was measured by trained medical staff, resulting in high quality (Arnold et al. 2017). An extensive occupational history of each participant was carried out via interview.

Although the cohort was quite large, unexpectedly few incident events were observed in the 5-year FU period indicated by the large confidence intervals of the risk estimates. Thus, the analyses lacked statistical power to find effect estimates. Healthy worker effect is possible, which means that the effects seen could be an underestimation (Baillargeon 2001). Although loss to FU was under 5% for the time to event CVD and diabetes analysis there were several missing values for arterial stiffness due to a lack of measuring devices at some point in the study. In addition, in some participants the elasticity of the vessel was so low that it could not be measured properly and only be labelled as ‘very stiff ‘. This could also lead to an underestimation of the effect. For the present analysis, we looked at working hours at the current job and did not include past jobs, which could possibly bias the results. The same applies to the fact that a number of participants (*n* = 1114) had missing values for the working time variable.

### Future research and recommendations

The 10-year FU of GHS will most likely result in more CVD events and occurrence of diabetes. The fact there was a significant increase in SI is promising. Further studies are needed to confirm the results on working hours and arterial stiffness. Upcoming (Lunde et al. 2020) and future results from occupational cohorts will help to evaluate the significance of arterial stiffness for occupational risk factors and preventive occupational health in general and long working hours in particular. Continued observation should also provide more information regarding the potential differences in long working hours between men and women.

In general, more attention should be paid to the management of cardiometabolic risk factors for those working long hours. Work organisation should facilitate regular and effective breaks. Health awareness on the part of the working population is also important to ensure work-life balance.

## Summary of results

Long working hours were only associated with arterial stiffness but not with incident CVD or diabetes in the 5-year follow-up of a population-based cohort. Future research is needed regarding longer follow-up and the potential of measuring arterial stiffness in preventive occupational health.

## Supplementary Information

Below is the link to the electronic supplementary material.

Supplementary file1 (DOCX 47 KB)
