# Supplementary material for: Long working hours and risk of cardiovascular outcomes and diabetes type II: five-year follow-up of the Gutenberg Health Study (GHS)
Source: Int Arch Occup Environ Health. 2021 Nov 12;95(1):303–12. doi: 10.1007/s00420-021-01786-9 (PMC8755657; doi:10.1007/s00420-021-01786-9)
Supplement: Supplementary file 1 — Supplementary file1 (DOCX 47 KB) [file 420_2021_1786_MOESM1_ESM.docx]

**Table 1: Sample characteristics of the analysis sample of the Gutenberg Health Study (GHS)**

**Table 1a: Characteristics by sex (n= 7241)**

|  | All  (n=7241) | Women  (n=3332) | Men  (n=3910) | p for trend |
| --- | --- | --- | --- | --- |
| Age [y] (mean±SD) | 48.3±7.6 | 48.1±7.4 | 48.6±7.7 | * |
| *General* |  |  |  |  |
| Qualification for university | 3445 (47.6%) | 1433 (43.0%) | 2012 (51.5%) | n.a. |
| SES (mean±SD) | 14.07±4.20 | 13.53±3.92 | 14.53±4.38 | *** |
| *Anthropometrics* |  |  |  |  |
| WHtR (mean±SD) | 0.54±0.08 | 0.53±0.08 | 0.55±0.07 | *** |
| Still regular period | - | 1682 (50.6%) | - | - |
| Menopausal age [y]  (mean±SD) | - | 46.82±6.31 | - | - |
| *Life style* |  |  |  |  |
| Smoking | 1731 (23.9%) | 788 (23.6%) | 943 (24.1%) | n.sig |
| -pack-years (median Q1/Q3) | 0.2 (0/3.6) | 0.1 (0/2.7) | 0.5 (0/4.3) | *** |
| Alcohol per day [g] (median (Q1/Q3)) | 5.0 (0/16.9) | 0 (0/9.4) | 8.4 (0/22.0) | *** |
| -Intake above tolerable limit | 1698 (23.5%) | 734 (22.0%) | 964 (24.7%) | n.a. |
| Activity score (mean±SD) | 8.4 ±3.4 | 8.1±3.0 | 8.7±3.7 | *** |
| *Occupational factors* |  |  |  |  |
| Years at current work place | 14.05±10.44 | 13.04±10.25 | 14.90±10.53 | *** |
| Managerial position | 1085 (15.0%) | 287 (8.6%) | 798 (20.4%) | *** |
| Self-employed | 1059 (14.6%) | 378 (11.4%) | 681 (17.4%) | *** |
| Employee | 6178 (85.4%) | 2951 (88.6%) | 3227 (82.6%) | *** |
| Full-time work | 5591 (77.2%) | 1821 (54.7%) | 3770 (96.4%) | *** |
| Part time employment | 1650 (22.8%) | 1511 (45.3%) | 139 (3.6%) | *** |
| Night shift | 964 (13.3%) | 254 (7.6%) | 710 (18.2%) | *** |
| Low job complexity° | 261 (3.6%) | 183 (5.5%) | 78 (2.0%) | *** |
| Medium job complexity° | 3289 (45.4%) | 1808 (54.3%) | 1481 (37.9%) | *** |
| High job complexity° | 1524 (21.0%) | 603 (18.1%) | 921 (23.6%) | *** |
| Very high job complexity° | 2167 (29.9%) | 738 (22.1%) | 1429 (36.6%) | *** |
| Working time total [h/w] (mean±SD) | 40.8±13.0 | 34.5±12.8 | 46.2±10.6 | *** |
| (median (Q1/Q3)) | 41 (35/48) | 36 (24/42) | 45 (40/50) | *** |
| Fixed working time [h/w] (mean±SD) | 36.9±11.3 | 31.4±11.1 | 41.6±9.2 | *** |
| Overtime [h/w] (mean±SD) | 3.9±6.1 | 3.10±5.29 | 4.63±6.56 | *** |
| Overtime>20% | 1265 (17.6%) | 451 (13.7%) | 814 (20.9%) | *** |
| *CVRF* |  |  |  |  |
| Obesity | 1607 (22.2%) | 663 (19.9%) | 944 (24.1%) | *** |
| FH of MI or Stroke | 2358 (32.6%) | 1143 (34.3%) | 1215 (31.1%) | *** |
| *S*tiffness-Index [m/s]  (mean±SD) | 7.25±2.13 | 6.39±1.57 | 7.95±2.27 | *** |
|  |  |  |  |  |

**Table 1b: Characteristics by total working time (n=7241)**

|  | All (n=7241) | ≤40 h/w]  (n=3459) | 41-54 h/w (n=2906) | ≥55 h/w (n=876) | p for trend |
| --- | --- | --- | --- | --- | --- |
| Sex (Women) | 3332 (46.0%) | 2269 (65.6%) | 899 (30.9%) | 164 (18.7%) | *** |
| Age [y] (mean±SD) | 48.3±7.6 | 48.6±7.5 | 47.9±7.6 | 49.0±7.7 | n.sig |
| *General* |  |  |  |  |  |
| Qualification for university | 3445 (47.6%) | 1410 (40.8%) | 1558 (53.6%) | 477 (54.5%) | n.a. |
| SES (mean±SD) | 14.07±4.20 | 13.05±4.06 | 14.85±4.07 | 15.53±4.19 | *** |
| *Anthropometric* |  |  |  |  |  |
| WHtR (mean±SD) | 0.54±0.08 | 0.54±0.08 | 0.54±0.07 | 0.55±0.07 | ** |
| *Life style* |  |  |  |  |  |
| Smoking | 1731 (23.9%) | 829 (24.0%) | 673 (23.2%) | 229 (26.1%) | n.sig. |
| -pack-years (median (Q1,Q3)) | 0.2 (0/3.6) | 0.1 (0/3.3) | 0.3 (0/3.5) | 0.4 (0/5.2) | * |
| Alcohol per day (g) (median (Q1,Q3)) | 5.0 (0/16.8) | 2.5 (0/12.6) | 5.6 (0/18.9) | 7.54 (0/20.6) | *** |
| -Intake above tolerable limit | 1698 (23.5%) | 768 (22.2%) | 708 (24.4%) | 222 (25.3%) | n.a. |
| Activity score (SQUASH) (mean±SD) | 8.4±3.4 | 8.0±3.2 | 8.5±3.3 | 9.7±4.3 | *** |
| *Occupational factors* |  |  |  |  |  |
| Years at current work place | 14.1±10.4 | 13.5±10.5 | 14.4±10.2 | 15.0±10.9 | *** |
| Managerial Position | 1085 (15.0%) | 228 (6.6%) | 557 (19.2%) | 300 (34.2%) | *** |
| Self-employed | 1059 (14.6%) | 356 (10.3%) | 295 (10.2%) | 408 (46.6%) | *** |
| Employee | 6178 (85.4%) | 3100 (89.7%) | 2611 (89.8%) | 467 (53.4%) | *** |
| Full-time | 5591 (77.2%) | 1857 (53.7%) | 2866 (98.6%) | 868 (99.1%) | *** |
| Part time employment | 1650 (22.8%) | 1602 (46.3%) | 40 (1.4%) | 8 (0.9%) | *** |
| Night shift | 964 (13.3%) | 328 (9.5%) | 412 (14.2%) | 224 (25.6%) | *** |
| Low job complexity° | 261 (3.6%) | 218 (6.3%) | 34 (1.2%) | 9 (1.0%) | *** |
| Medium job complexity° | 3289 (45.4%) | 1921 (55.5%) | 1099 (37.8%) | 269 (30.7%) | *** |
| High job complexity° | 1524 (21.0%) | 604 (17.5%) | 724 (24.9%) | 196 (22.4%) | *** |
| Very high job complexity° | 2167 (29.9%) | 716 (20.7%) | 1049 (36.1%) | 402 (45.9%) | *** |
| Working time total [h/w] (mean±SD) | 40.8±13.0 | 31.1±9.0 | 45.7±3.4 | 63.1±9.6 | *** |
| (median (Q1/Q3)) | 41 (35/48) | 34 (24/39.5) | 45 (43/50) | 60 (57/65) |  |
| Fixed working time [h/w]  (mean±SD) | 36.9±11.3 | 29.8±9.3 | 40.6±3.8 | 52.7±13.2 | *** |
| Overtime [h/w] (mean±SD) | 3.9±6.1 | 1.3±2.7 | 5.1±4.0 | 10.5±12.0 | *** |
| Overtime>20% | 1265 (17.6%) | 201 (5.9%) | 692 (23.9%) | 372 (43.7%) | *** |
| *CVRF* |  |  |  |  |  |
| Obesity | 1607 (22.2%) | 760 (22.0%) | 627 (21.6%) | 220 (25.1%) | n.sig |
| FH of MI or Stroke | 2358 (32.6%) | 1156 (33.4%) | 926 (31.9%) | 276 (31.5%) | n.sig |
| *S*tiffness-Index [m/s] (mean±SD) | 7.25±2.13 | 6.94±.95 | 7.44±2.20 | 7.82±2.38 | *** |

Legend: °= from KldB2010, n.sig=non significant, n.a.=not applicable,*= < 0.05, **= <0.001,

***= <0.0001, WHtR= Waist to height ratio, SES= social economic status, h/w= hours per week, CVRF=cardiovascular risk factors, FH=family history, MI=myocardial infarction

|  | **n at baseline** | **n at FU** | **Events within FU period** | **Censored events** | **Person years (py)** | **Incidence rate per 1000 py (95%Cl)** |
| --- | --- | --- | --- | --- | --- | --- |
| **CVD** |  |  |  |  |  |  |
| All participants | 7241 | 6816 | 122 | 6694 | 33523 | 3.64 (3.02-4.35) |
| ≤40 h/w | 3459 | 3252 | 51 | 3201 | 16007 | 3.19 (2.37-4.19) |
| 41-54 h/w | 2906 | 2765 | 53 | 2712 | 13614 | 3.89 (2.92-5.10) |
| ≥55 h/w | 876 | 799 | 18 | 781 | 3902 | 4.61 (2.73-7.30) |
| Men | 3909 | 3625 | 98 | 3527 | 17726 | 5.53 (4.49-6.74) |
| ≤40 h/w | 1190 | 1081 | 34 | 1047 | 5251 | 6.47 (4.48-9.05) |
| 41-54 h/w | 2007 | 1898 | 48 | 1850 | 9326 | 5.15 (3.79-6.82) |
| ≥55 h/w | 712 | 646 | 16 | 630 | 3149 | 5.08 (2.90-8.23) |
| Women | 3332 | 3191 | 24 | 3167 | 15797 | 1.52 (0.97-2.26) |
| ≤40 h/w | 2269 | 2171 | 17 | 2154 | 10755 | 1.58 (0.92-2.53) |
| 41-54 h/w | 899 | 867 | 5 | 862 | 4289 | 1.17 (0.38-2.72) |
| ≥55 h/w | 164 | 153 | 2 | 151 | 753 | 2.66 (0.32-9.60) |
| **Diabetes** |  |  |  |  |  |  |
| All participants | 7241 | 6613 | 126 | 6487 | 32462 | 3.88 (3.23-4.62) |
| ≤40 h/w | 3459 | 3157 | 62 | 3095 | 15511 | 4.00 (3.06-5.12) |
| 41-54 h/w | 2906 | 2669 | 48 | 2621 | 13116 | 3.66 (2.70-4.85) |
| ≥ 55 h/w | 876 | 787 | 16 | 771 | 3835 | 4.17 (2.38-6.78) |
| Men | 3909 | 3504 | 80 | 3424 | 17129 | 4.67 (3.70-5.81) |
| ≤40 h/w | 1190 | 1039 | 30 | 1009 | 5055 | 5.93 (4.00-8.47) |
| 41-54 h/w | 2007 | 1833 | 35 | 1798 | 8997 | 3.89 (2.71-5.41) |
| ≥ 55 h/w | 712 | 632 | 15 | 617 | 3078 | 4.87 (2.72-8.04) |
| Women | 3332 | 3109 | 46 | 3063 | 15333 | 3.00 (2.20-4.00) |
| ≤40 h/w | 2269 | 2118 | 32 | 2086 | 10456 | 3.06 (2.10-4.32) |
| 41-54 h/w | 899 | 836 | 13 | 823 | 4119 | 3.16 (1.68-5.40) |
| ≥55 h/w | 164 | 155 | 1 | 154 | 757 | 1.32 (0.03-7.36) |

**Table 2: Associations of working time with incidence of CVD and diabetes (time to event). Number of events, censored events, person-time and unadjusted incidence rates.**

**Table 3: Beta coefficients and 95% confidence intervals (CI) of arterial stiffness according to weekly working hours (h/w)**

|  | **Beta coefficients and 95% confidence intervals (CI) of arterial stiffness according to weekly working hours** | | |
| --- | --- | --- | --- |
|  | ≤40 h/w (Reference) | 41–54 h/w | ≥55 h/w |
| Model 0 | 1 | 0.52 (0.35-0.68) | 0.90 (0.66-1.15) |
| Model 1 | 1 | 0.04 (-0.13-0.21) | 0.22 (-0.03-0.47) |
| Model 2 | 1 | 0.05 (-0.12-0.23) | 0.22 (-0.04-0.47) |
| Model 3 | 1 | 0.07 (-0.10-0.24) | 0.23 (-0.03-0.47) |
| Model 4 | 1 | 0.11 (-0.07-0.28) | 0.32 (0.07-0.58) |
| Model 5 | 1 | 0.11 (-0.07-0.28) | 0.28 (0.02-0.54) |

Legend: Model 0: crude model (exposure only); Model 1: sex and age; Model 2: model 1 plus night shift, managerial/supervisor position, years at current work place; Model 3: model 1 plus waist to height ratio, smoking status, pack-years, alcohol consumption, physical activity, menopausal status; Model 4: model 1 plus SES; Model 5: all confounders

**Supplementary tables**

**Table S 1: Hazard ratios (HR) and 95% confidence intervals (CI) for incidence of CVD according to weekly working hours**

|  | **Hazard ratios (HR) and 95% confidence intervals (CI) for incidence of CVD according to weekly working hours** | | |
| --- | --- | --- | --- |
|  | ≤40 h/w (Reference) | 41–54 h/w | ≥55 h/w |
| Model 0 | 1 | 1.19 (0.81-1.76) | 1.45 (0.85-2.47) |
| Model 1 | 1 | 0.86 (0.58-1.29) | 0.86 (0.49-1.50) |
| Model 2 | 1 | 0.90 (0.60-1.36) | 0.95 (0.52-1.71) |
| Model 3 | 1 | 0.88 (0.59-1.32) | 0.82 (0.47-1.43) |
| Model 4 | 1 | 0.92 (0.61-1.37) | 0.93 (0.53-1.63) |
| Model 5 | 1 | 0.90 (0.60-1.37) | 0.88 (0.48-1.61) |

Legend: Model 0: crude model (exposure only), Model 1: sex and age, Model 2: model 1 plus night shift, managerial/supervisor position, years at current work place, Model 3: model 1 plus waist to height ratio, smoking status, pack-years, alcohol consumption, physical activity, menopausal status Model 4: model 1 plus SES, Model 5: all confounders

**Table S 2: Hazard ratios (HR) and 95% confidence intervals (CI) for incidence of diabetes according to weekly working hours**

|  | **Hazard ratios (HR) and 95% confidence intervals (CI) for incidence of diabetes according to weekly working hours** | | |
| --- | --- | --- | --- |
|  | ≤40 h/w (Reference) | 41–54 h/w | ≥55 h/w |
| Model 0 | 1 | 0.94 (0.64-1.38) | 1.08 (0.62-1.87) |
| Model 1 | 1 | 0.82 (0.54-1.23) | 0.84 (0.48-1.49) |
| Model 2 | 1 | 0.85 (0.56-1.28) | 0.93 (0.51-1.68) |
| Model 3 | 1 | 0.85 (0.56-1.28) | 0.81 (0.46-1.43) |
| Model 4 | 1 | 0.91 (0.60-1.38) | 0.98 (0.55-1.76) |
| Model 5 | 1 | 0.90 (0.59-1.38) | 0.91 (0.49-1.69) |

Legend: Model 0: crude model (exposure only), Model 1: sex and age, Model 2: model 1 plus night shift, managerial/supervisor position, years at current work place Model 3: model 1 plus waist to height ratio, smoking status, pack-years, alcohol consumption, physical activity, menopausal status, Model 4: model 1 plus SES, Model 5: all confounders

**Table S 3: Beta coefficients and 95% confidence intervals (CI) of arterial stiffness according to weekly working hours with SI at t_0_ as offset variable**

|  | **Beta coefficients and 95% confidence intervals (CI) of arterial stiffness according to weekly working hours** | | |
| --- | --- | --- | --- |
|  | ≤40 h/w (Reference) | 41–54 h/w | ≥55 h/w |
| Model 0 | 1 | 0.38 (0.21-0.55) | 0.65 (0.40-0.90) |
| Model 1 | 1 | 0.05 (-0.13-0.23) | 0.20 (-0.06-0.47) |
| Model 2 | 1 | 0.06 (-0.13-0.24) | 0.21 (-0.06-0.48) |
| Model 3 | 1 | 0.07 (-0.11-0.25) | 0.22 (-0.05-0.48) |
| Model 4 | 1 | 0.08 (-0.14-0.27) | 0.25 (-0.02-0.52) |
| Model 5 | 1 | 0.10 (-0.09-0.28) | 0.26 (-0.02- 0.53) |

Legend: Model 0: crude model (exposure only); Model 1: sex and age; Model 2: model 1 plus night shift, managerial/supervisor position, years at current work place; Model 3: model 1 plus waist to height ratio, smoking status, pack-years, alcohol consumption, physical activity, menopausal status; Model 4: model 1 plus SES; Model 5: all confounders
